# Small Waists

**Published:** 1883-06

**Authors:** 


### SMALL WAISTS.

Again and again it has been asserted that small waists are not beautiful, according to the sculptor's idea, else Venus would not have been represented as the plump goddess familiar to all lovers of statuary. Yet women insist on wearing corsets to form a figure supposed to be pleasing to men. Let him who admires the piece of pipe that does duty for a human body picture to himself the wasted form and seamed skin. Most women, from long custom of wearing these stays, are really unaware how much they are hampered and restricted. A girl of twenty, intended by nature to be one of her finest specimens, gravely assures one her stays are not tight, being exactly the same size as those she was first put into, not perceiving her condemnation in the fact that she has since grown five inches in height and two in shoulder breadth. Her stays are not too tight, because the constant pressure has prevented the natural development of heart and lung space. The dainty waists of the poets is precisely that inflexible slimness that is destroyed by stays. The form resulting from them is not slim, but a piece of pipe, and as inflexible. But while endeavoring to make clear the outrage upon practical good sense and sense of beauty, it is necessary to understand and admit the whole state of the case. A reason, if not a necessity, for some sort of corset may be found when the form is very redundant; this, however, cannot be with the very young and slight, but all that necessity could demand, and that practical good sense and fitness would concede, could be found in a strong, elastic kind of jersey, sufficiently strong, and even stiff, under the bust to support it and sufficiently elastic at the sides and back to injure no organs and impede no functions.

---

House Plants have very little effect on the occupants of a room where they are; they consume carbonic acid gas and give out oxygen, but not in quantities to make any appreciable difference in the healthfulness of the atmosphere. But care should be observed in the use of fertilizers; the air of a room may be contaminated by emanations from some kinds of house-plant fertilizers, unless the room is kept constantly ventilated; that is, unless the ventilation is sufficient to change the air completely three or four times in twenty-four hours. The plants should be set in earth that has been thoroughly enriched with stable manure, and long enough to have become combined with it; by this combination the earth disinfects the manure by absorbing and neutralizing any noxious gases that may exist.

